# Proliferating CLL cells express high levels of CXCR4 and CD5

**DOI:** 10.1002/hem3.70064

**Published:** 2024-12-17

**Authors:** Daniel Friedman, Drshika P. Mehtani, Jennifer B. Vidler, Piers E. M. Patten, Robbert Hoogeboom

**Affiliations:** ^1^ Department of Haemato‐Oncology Comprehensive Cancer Centre, King's College London London UK; ^2^ Department of Haematological Medicine King's College Hospital London UK

## Abstract

Chronic lymphocytic leukemia (CLL) is an incurable progressive malignancy of CD5^+^ B cells with a birth rate between 0.1% and 1% of the entire clone per day. However, the phenotype and functional characteristics of proliferating CLL cells remain incompletely understood. Here, we stained peripheral blood CLL cells for ki67 and DNA content and found that CLL cells in G1‐phase have a CXCR4^lo^CD5^hi^ phenotype, while CLL cells in S/G2/M‐phase express high levels of both CXCR4 and CD5. Induction of proliferation in vitro using CD40L stimulation results in high ki67 levels in CXCR4^lo^CD5^hi^ cells with CXCR4 expression increasing as CLL cells progress through S and G2/M‐phases, while CXCR4^hi^CD5^lo^ CLL cells remained quiescent. Dye dilution experiments revealed an accumulation of Ki67^hi^‐divided cells in the CXCR4^hi^CD5^hi^ fraction. In Eµ‐TCL1 transgenic mice, the CXCR4^hi^CD5^hi^ fraction expressed high levels of ki67 and was expanded in enlarged spleens of diseased animals. Human peripheral blood CXCR4^hi^CD5^hi^ CLL cells express increased levels of IgM and the chemokine receptors CCR7 and CXCR5 and migrate efficiently toward CCL21. We found higher levels of CXCR4 in patients with progressive disease and the CXCR4^hi^CD5^hi^ fraction was expanded upon clinical relapse. Thus, this study defines the phenotype and functional characteristics of dividing CLL cells identifying a novel subclonal population that underlies CLL pathogenesis and may drive clinical outcomes.

## Graphical abstract

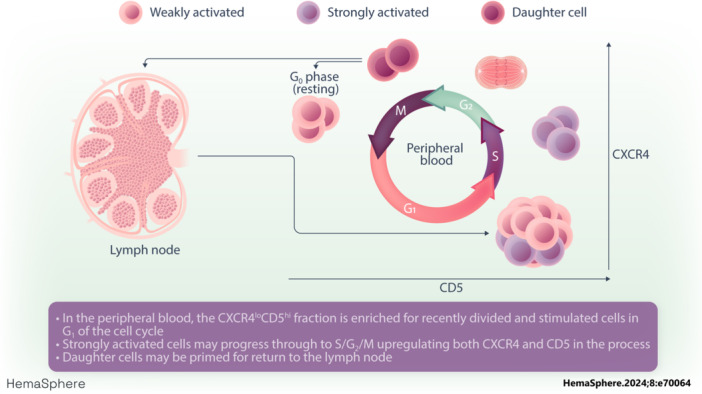

## INTRODUCTION

Chronic lymphocytic leukemia (CLL) is characterized by an expansion of malignant CD5^+^ B cells in the peripheral blood (PB), bone marrow (BM), and secondary lymphoid tissues. CLL cells proliferate substantially with between 0.1% and 1% of the clone renewed per day.^1^ In the clinic, CLL patients display a heterogeneous course. CLL cases expressing B cell receptors (BCRs) with unmutated *IGHV* (U‐CLL) have a worse prognosis and more frequently require treatment, while CLL with mutated *IGHV* (M‐CLL) genes may have stable disease for years, reflecting higher net CLL cell birthrates in U‐CLL patients.^2^, ^3^

In vivo deuterium labeling revealed that proliferation rates of CLL cells are highest in the lymph nodes (LNs), where CLL cells display more prominent BCR activation and resident immune and stromal cells present key stimulatory ligands, such as antigen and CD40 ligand (CD40L).^4^, ^5^ Following stimulation and proliferation in LNs, CLL cells may emigrate to the PB, where cells may acquire a quiescent state until either tissue re‐entry and re‐stimulation or death ensues. While the PB compartment is largely deemed quiescent, the discovery of subpopulations of newly born and quiescent cells in the PB indicates a spectrum of cell states among circulating CLL cells in the periphery.^6^ At one end are recently proliferated and activated LN emigrants, expressing low levels of the chemokine C‐X‐C motif receptor 4 (CXCR4) and high levels of CD5, often used as a proxy for proliferating cells in LNs.^7^, ^8^, ^9^ At the other end, the fraction of cells with high CXCR4 and low CD5 is enriched in quiescent cells. However, a recent study integrating bulk and single‐cell RNA sequencing of LN and PB CLL cells identified three major cell states in both compartments: quiescent, activated, and proliferating,^10^ suggesting that the current CXCR4/CD5 model may not capture all cell states.

Identifying which cells proliferate is important for developing therapeutic strategies that target the most relevant cells, and we, therefore, set out to identify and functionally characterize actively dividing cells from the PB of CLL patients. We find that PB CLL cells in the S, G2, and M phases uniformly express high levels of CXCR4 and CD5. These CXCR4^hi^CD5^hi^ cells are enriched for highly proliferative cells upon stimulation in vitro and have elevated levels of chemokine receptors, potentially facilitating efficient migration in response to LN homing signals. Finally, we observed increased levels of CXCR4 and CD5 in PB CLL samples at relapse. Together, this study provides novel insights into the phenotype and nature of proliferating cells in CLL as well as new starting points to develop biomarkers for disease progression and response to therapies that target proliferative signals.

## MATERIALS AND METHODS

### Patients and samples

This research was supported by material from the King's College Denmark Hill Haematology Biobank (18/NE/0141). Cryopreserved PB samples were obtained from CLL patients with written informed consent in accordance with the Declaration of Helsinki. PB and LN fine needle aspirates were taken as previously described.^11^ Supporting Information S1: Table 1 lists the patient details of the samples used in this study.

### Mice

Animal experiments were approved by the UK Home Office. BM, spleen, and PB were harvested from aged *Eµ‐TCL1* mice (>10 months). Mice harboring <50% CD19^+^ CD5^+^ splenic lymphocytes were excluded from the study. Single‐cell suspensions from tissue were obtained by mechanical disruption and passing cells through 40 µm cell strainers (Corning). Red blood cells were lysed in blood and spleen samples using RBC‐lysis buffer (Biolegend).

### In vitro proliferation assays

Primary CLL cells were cultured for up to 9 days in Iscove's Modified Dulbecco's Medium (IMDM; Gibco) supplemented with 10% fetal bovine serum (Gibco), 1% Penicillin/Streptomycin, and 1% L‐glutamine (both Sigma). Where indicated, CLL PBMCs were cocultured on irradiated NIH3T3 fibroblasts stably transfected with CD40 ligand or parental cells. To track proliferation histories, PBMCs (10 × 10^6^/mL) were labeled with 0.5 µM CMFDA (ThermoFisher) in phosphate‐buffered saline (PBS) for 20 min at 37°C prior to seeding.

### Flow cytometry

Human PBMCs were treated with Fc blocking solution (Human TruStain FcX; Biolegend) and stained with the following fluorescent antibodies from Biolegend (unless otherwise stated): anti‐CD19 (HIB19), anti‐CD5 (L17F12), anti‐CXCR4 (12G5), anti‐CCR7 (G043HD), anti‐CXCR5 (J252D4), anti‐IgM (MHM‐88), anti‐CD49d (9F10), anti‐AID (BD Biosciences, EK2‐5G9), anti‐Ki67 (11F6), and anti‐NFAT2 (7A6). Mouse primary cells were treated with anti‐mouse CD16/32 (Biolegend) and stained with fluorescent antibodies: anti‐CD19 (6D5), anti‐CD5 (53‐7.3), anti‐CXCR4 (L276F12), and anti‐Ki67 (11F6). A fixable viability dye (eBioscience) was used to exclude dead cells. Cells were fixed with 4% PFA and washed with PBS prior to acquisition. For intracellular staining, cells were fixed and permeabilized using the FoxP3/Transcription factor staining kit (eBioscience) and stained overnight for Ki67 at 4°C. To discriminate between cell cycle stages, permeabilized cells were treated with 100 µg/mL RNase A (Sigma) for 15 min at 37°C followed by labeling with 0.5 µg/mL 4′,6‐diamidino‐2‐phenylindole (DAPI) (Sigma) for 10 min at room temperature. A minimum of 300,000 CD19^+^ CD5^+^ cells were acquired per patient on a BD LSR Fortessa and data were analyzed using Flowjo software (TreeStar, V10). For imaging flow cytometry, cells were stained as above and analyzed on an ImagestreamX MK‐II (Amnis). Brightfield and fluorescent images were captured using ×40 zoom, and data were analyzed using IDEAS (Merck). Cell debris and doublets were excluded prior to analysis.

### Migration assays

Migration assays were performed using 5‐μm pore polycarbonate transwell inserts in 24‐well plates (Corning). Transwell filters were coated overnight with 2.5 µg/mL ICAM‐1 (R&D systems) in PBS and blocked with 2% BSA/PBS for 2 h; 0.5% BSA plus 0.1/1 µg/mL CCL21 (R&D systems) was added to basolateral chambers, and 0.5 × 10^6^ PBMCs were transferred into the apical chambers and incubated for 2 h at 37°C/5% carbon dioxide. Time‐matched controls were incubated in ICAM‐1‐coated wells in parallel with the migration assays. After 2 h, 40 mM EDTA/PBS was added to each well/filter followed by staining with fluorescent antibodies against CD19, CD5, and CXCR4 as described above.

### Statistical analysis

Statistical analyses were carried out using GraphPad Prism (version 10). Shapiro–Wilk normality tests were used to evaluate the distribution of values. Data are presented as means±standard deviation.

## RESULTS

### Proliferating CLL cells in the blood express high levels of CXCR4 and CD5

**Figure 1:**
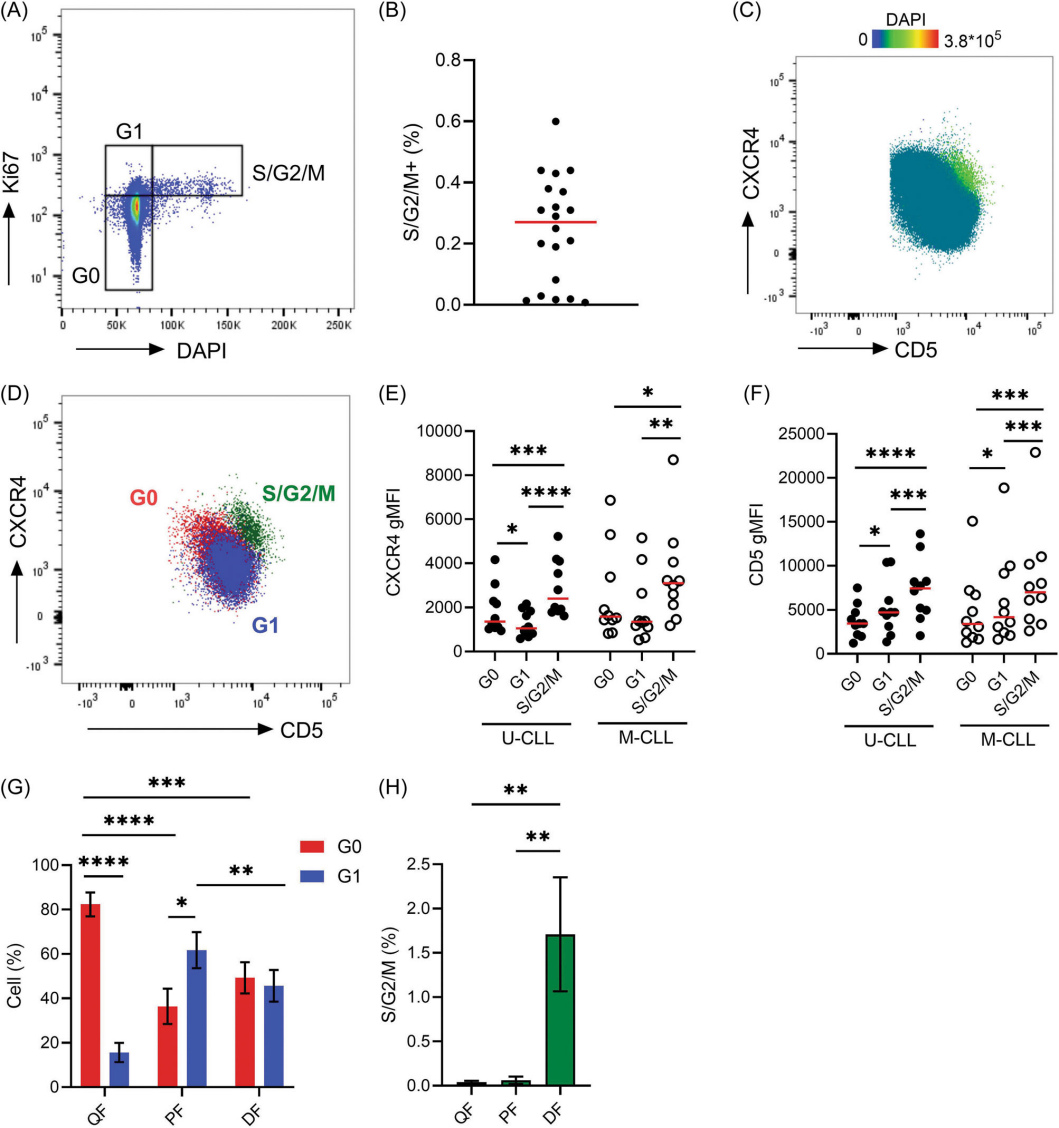
**Proliferating CLL cells in the blood express high levels of CXCR4 and CD5**. **(A)** Representative scatter plot of Ki67 and DAPI staining on CLL cells highlighting cell fractions in G0, G1, and S/G2/M phases. **(B)** Quantification of the percentage of cells in S/G2/M phases. *n* = 20 **(C)** CXCR4 versus CD5 scatter plot overlaid with cells in S/G2/M‐phase, which localize to the CXCR4^hi^CD5^hi^ fraction. DAPI mean fluorescence intensities are displayed as a heat map scale. **(D)** Representative scatter plot showing CXCR4 and CD5 expression on G0, G1, and S/G2/M fractions as gated in **(A)**. Quantification of **(E)** CXCR4 and **(F)** CD5 gMFIs on cells in G0, G1, and S/G2/M phases for both M‐ and U‐CLL patients. Each data point represents a single patient, *n* = 10. **(G)** The percentage of cells in the quiescent fraction (QF), proliferated fraction (PF), and dividing fraction (DF) were quantified for the G0, G1, and (**H**) S/G2/M populations. gMFI, geometric mean fluorescence intensities. Statistical significance of data was calculated using repeated measures (RM) one‐way ANOVA with Tukey's multiple comparisons. *p < 0.05, **p < 0.01, ***p < 0.001, ****p < 0.0001.

The low frequency of proliferating cells (~0.1%–1% of the leukemic clone) has made it challenging to identify and characterize actively dividing cells in the periphery. Here, we stained PB samples from U‐CLL and M‐CLL patients for DNA content and Ki67 expression to distinguish cells in G0 (2*n* DNA, ki67^lo^), G1 (2*n* DNA, ki67^hi^), and S/G2/M (4n DNA, Ki67^hi^) phases (Figure 1A). Consistently, we detected a fraction of cells in S/G2/M phases (0.24% ± 0.17%) in all 20 patients analyzed (Figure 1B and Supporting Information S1: Table 1).

In vivo deuterium labeling studies have identified that the CXCR4^lo^CD5^hi^ cell fraction is enriched in recently proliferated cells, while CXCR4^hi^CD5^lo^ phenotypes represent older quiescent CLL cells.^6^ To investigate which cell fraction contains actively dividing cells, we stained PB CLL cells for CXCR4, CD5, and DNA content. Surprisingly, cells with >2*n* DNA map to a fraction with high CXCR4 and high CD5 (Figure 1C), indicating that actively dividing CLL cells have a distinct surface phenotype. In agreement, we observed CXCR4^hi^CD5^hi^ cells in mitosis using imaging flow cytometry (Supporting Information S1: Figure 1a).

To interrogate the phenotypes of quiescent and proliferating CLL cells further, we overlayed the G0, G1, and S/G2/M fractions revealing distinct CXCR4 and CD5 expression profiles (Figure 1D) with lowest levels of CXCR4 on cells in G1 in U‐CLL and highest CXCR4 expression levels on cells in S/G2/M for both U‐CLL and M‐CLL (Figure 1E). CD5 expression increased as cells progressed through the cell cycle peaking in S/G2/M phases for both M‐CLL and U‐CLL cells (Figure 1F). Background autofluorescence may fluctuate during the cell cycle.^12^ However, changes in autofluorescence contributed negligibly to fluorescence intensities of surface markers (Supporting Information S1: Figure 1b).

Differential expression of CXCR4 and CD5 is widely used to distinguish the previously characterized quiescent CXCR4^hi^CD5^lo^ (QF) and recently proliferated CXCR4^lo^CD5^hi^ fraction (PF).^5^, ^13^, ^14^, ^15^ To examine the frequency of G0, G1, and S/G2/M cells within these fractions, 5% gates were drawn as previously described.^6^ An additional gate was drawn to capture the CXCR4^hi^CD5^hi^ subpopulation that we have here identified to harbor cells in the S/G2/M phase (dividing fraction [DF], Supporting Information S1: Figure 1c). The QF was enriched in G0 cells while the PF contained the highest levels of G1 cells (Figure 1G). The DF harbored the highest numbers of S/G2/M cells with a similar proportion of both G0 and G1 cells (Figure 1G,H). Altogether, these data redefine the current CXCR4/CD5 kinetics model, demonstrating that the PF is enriched for G1 cells while mitotic CLL cells express high levels of both CXCR4 and CD5.

### Modulation of CXCR4 expression in proliferating cells

**Figure 2:**
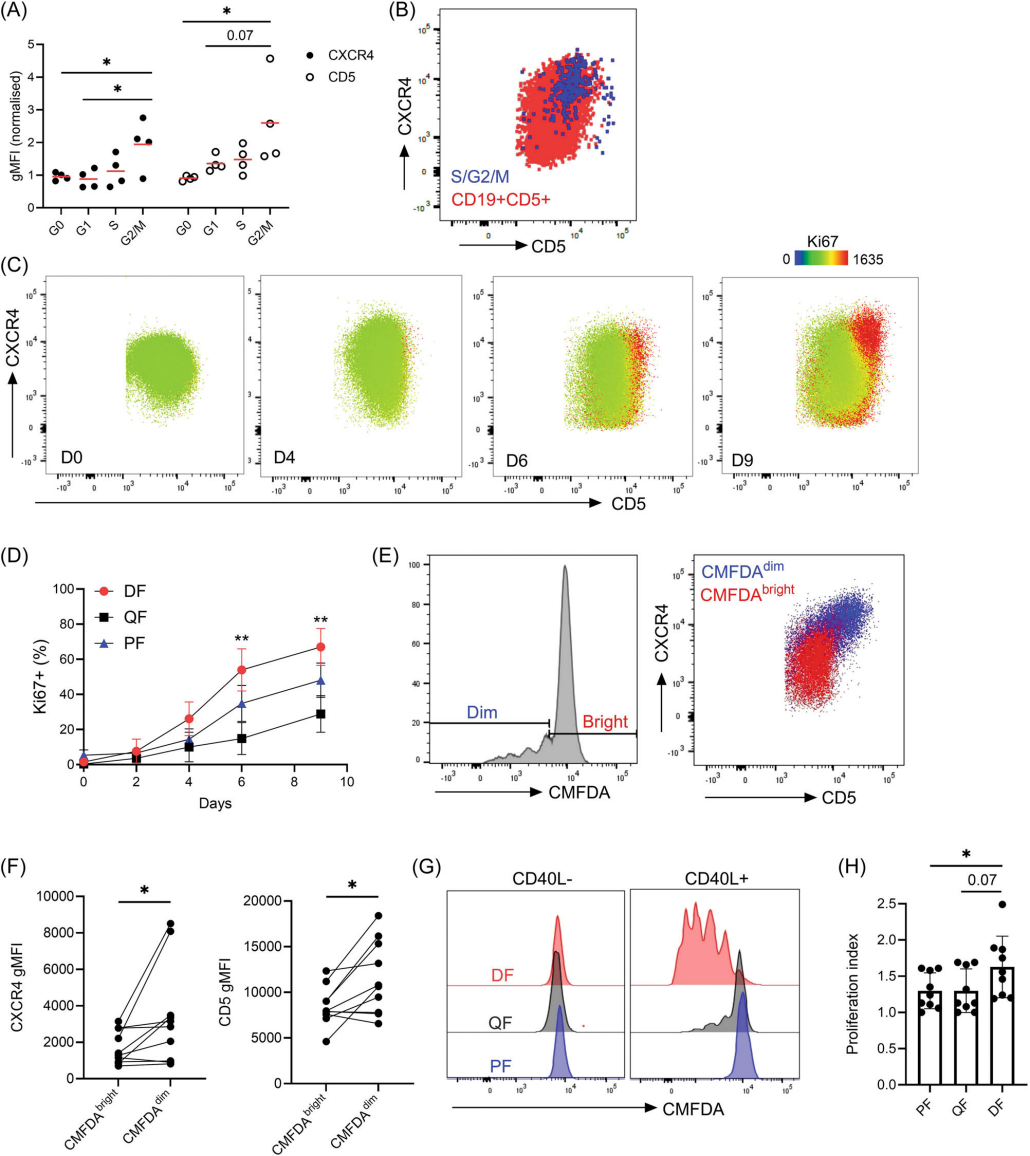
**CXCR4 and CD5 are upregulated when CLL cells progress through the cell cycle**. **(A)** Quantification of CXCR4 and CD5 expression levels of U‐CLL cells in G0, G1, and S/G2/M phases when stimulated on CD40L‐expressing fibroblasts after 6 days. (*n* = 4). Data were normalized to the bulk CXCR4/CD5 gMFI levels. **(B)** Representative CXCR4/CD5 scatter plot overlaid with the S/G2/M+ fraction from U‐CLL cells stimulated on CD40L‐expressing fibroblasts for 6 days. **(C)** Human U‐CLL cells were seeded on CD40L‐expressing fibroblasts for 9 days and samples were assessed for Ki67 expression on Days 0, 2, 4, 6, and 9. Representative scatter plots from a U‐CLL patient of CXCR4 and CD5 profiles from Days 0, 4, 6, and 9 are shown with Ki67 gMFIs overlaid as a heatmap statistic. **(D)** Frequency of Ki67+ cells in different cell fractions of U‐CLL cultured for 9 days (*n* = 8). **(E)** Divided (CMFDA^dim^) and nondivided U‐CLL cells (CMFDA^bright^) were separated (left panel) and their CXCR4/CD5 profiles overlaid (right panel). **(F)** Quantification of both CXCR4 and CD5 levels on both CMFDA^bright^ and CMFDA^dim^ populations from CD40L‐stimulated CLL cells after 9 days (*n* = 9). **(G)** Representative histogram plots from CMFDA‐stained CLL cells stimulated on CD40L‐expressing fibroblasts for 9 days. **(H)** Quantification of proliferation indices from CLL cells after 9 days of stimulation (*n* = 8). Data show mean ± SD. Statistical significance of data was calculated using an RM one‐way ANOVA with Tukey's multiple comparisons or a Friedman's test with Dunn's multiple comparisons *p < 0.05, **p < 0.01. ANOVA, analysis of variance; DF, dividing fraction; gMFI, geometric mean fluorescence intensities; PF, proliferated fraction; QF, quiescent fraction.

Our observation that G1 CLL cells express low levels of CXCR4 while S/G2/M cells have CXCR4^hi^CD5^hi^ phenotypes suggests that the surface phenotype of CLL cells may change when cells progress through the cell cycle. To investigate how CXCR4 and CD5 expression varies on cells in different phases of the cell cycle, proliferation of CLL cells was induced in vitro using CD40‐ligand (CD40L) expressing fibroblasts. CD40L expressing fibroblasts induce more robust and reliable proliferation in U‐CLL (Supporting Information S1: Figure 2a) and we focused on this subset of patients to model changes in CXCR4 and CD5. After 48 h on CD40L, when bulk ki67 levels were still low, CLL cells displayed significantly reduced surface CXCR4 expression, while CD5 levels remained unchanged. Contrastingly, in unstimulated cells, CXCR4 levels increased three‐fold while CD5 surface levels increased 1.3‐fold (Supporting Information S1: Figure 2b–d).

After 6 days of CD40L stimulation, we observed a stepwise increase in both CXCR4 and CD5 expression as cells progressed from G1 into S and G2/M phases (Figure 2A) and the S/G2/M subpopulation mapped to the DF (Figure 2B), mirroring our findings in freshly thawed PBMCs. Similar results were obtained when stimulating cells with CD40L, IL‐4, and IL‐21, a combination that induces more robust proliferation^16^ (Supporting Information S1: Figure 2e,f). To investigate if the quiescent, activated, and proliferating states observed in PB are recapitulated in in vitro proliferating CLL cells, we gated the QF, PF, and DF as previously described. From 6 days onwards of CD40 activation, when CLL cells are proliferating, the PF and the DF were significantly enriched with Ki67^+^ cells in CLL cell cultures, while ki67 expression remained low in the QF (Figure 2C,D). Likewise, in CD40L, IL‐4, and IL‐21‐stimulated cultures, we observed significant enrichment of ki67^+^ cells in the PF and DF, albeit earlier than with CD40L alone (Supporting Information S1: Figure 2g,h).

To examine and track the CXCR4 and CD5 phenotype of cells post‐cell division, cells were stained with the fluorescent dye CMFDA prior to seeding. After 9 days of CD40L stimulation, cell populations were subsequently split into fractions that had not proliferated (CMFDA^bright^) or had undergone at least one round of cell division (CMFDA^dim^). We observed significant upregulation of both CXCR4 and CD5 on the bulk CMFDA^dim^ fraction (Figure 2E,F). Consistent with this finding we observed significant enrichment of divided cells in the DF compared to the PF. Of note, differences between the DF and QF failed to reach statistical significance (*p* = 0.07) due to a small number of divided cells present in the QF (Figure 2G,H). Thus, our data demonstrates that while activating stimuli initially leads to a strong downregulation of CXCR4 reflecting cell entry into G1, it quickly resurfaces when cells enter S/G2/M phases, revealing an intricate relationship between activation, proliferation, and migration potential.

### CXCR4^hi^CD5^hi^ cells are highly proliferative in the TCL1 mouse model for CLL

**Figure 3:**
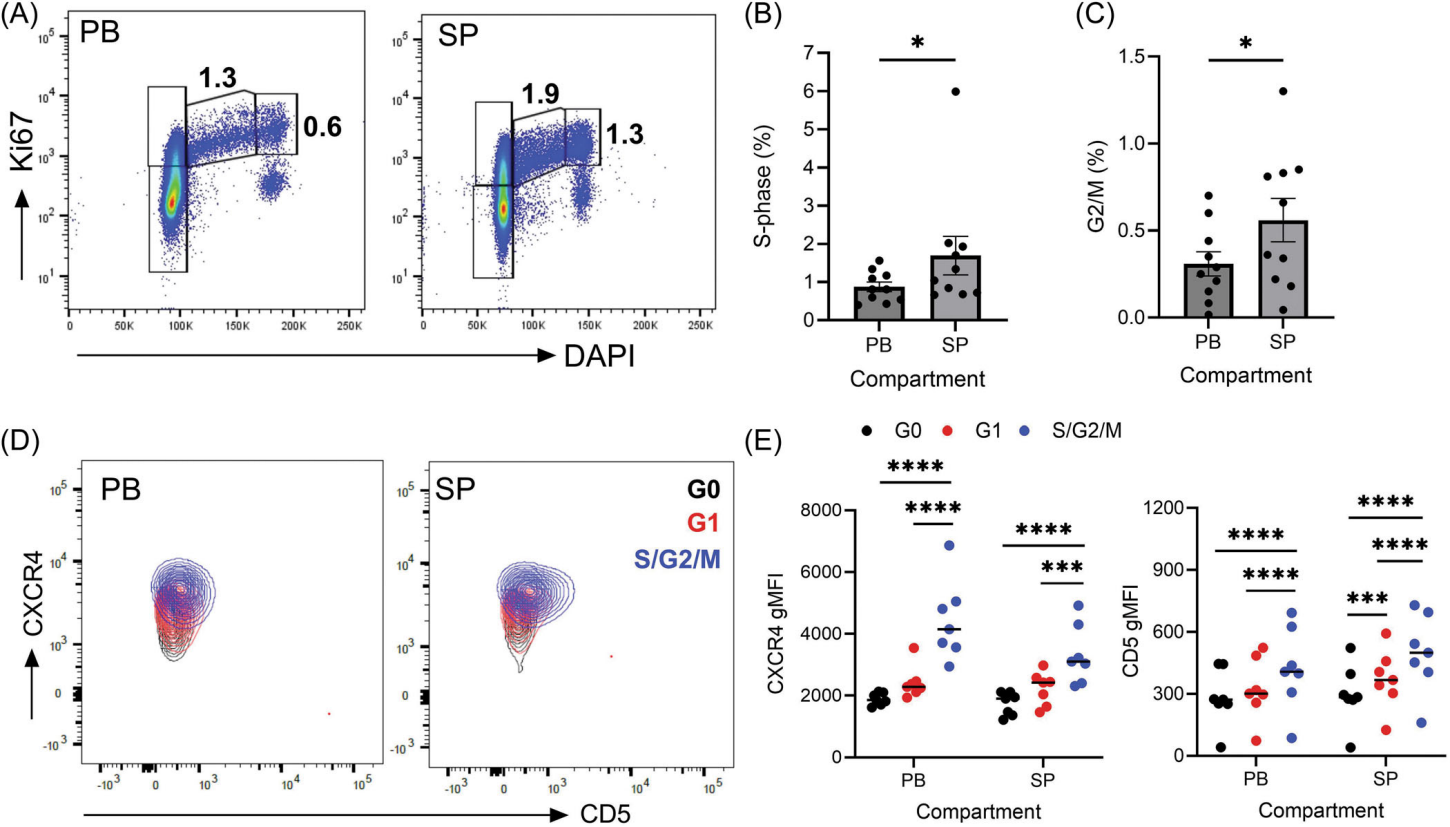
**CXCR4** ^**hi**^ **CD5** ^**hi**^ **cells are highly proliferative in the Eµ‐TCL1 mouse model for CLL**. **(A)** Representative scatter plots of Ki67 and DAPI staining of CD19^+^ CD5^+^ cells in the peripheral blood (PB) and spleen (SP) highlighting cell fractions in G0, G1, S, and G2/M phases. Ki67^lo^DAPI^hi^ populations were considered shadow doublets and excluded.^20^ **(B)** Quantification of the percentage of cells in S‐ and **(C)** G2/M phases between PB and SP compartments. (*n* = 9). (**D**) Representative contour plots showing CXCR4/CD5 expression on G0 (black), G1 (red), and S/G2/M (blue) fractions as gated in **(A)**. **(E)** Quantification of CXCR4 and CD5 gMFIs on cells in G0, G1 and S/G2/M phases from the PB and SP compartments. Each data point represents a single mouse (*n* = 7). Statistical significance of data was calculated using an RM one‐way ANOVA with Tukey's multiple comparisons. *p < 0.05, ***p < 0.001, ****p < 0.0001. ANOVA, analysis of variance; gMFI, geometric mean fluorescence intensities.

The transgenic *Eµ‐TCL1* mouse in which B cell‐directed TCL1 expression drives the development of a CLL‐like disease is a commonly used model for aggressive and high‐risk CLL^17^, ^18^, ^19^; however, the proliferating compartment in this model is poorly characterized. We found high levels of Ki67 in CD19^+^CD5^+^ murine leukemia cells from the PB and spleen while leukemic cells in the BM expressed significantly lower levels of ki67, suggesting that in this mouse model, proliferating cells are not sequestered in the BM compartment (Supporting Information S1: Figure 3a). Co‐staining PB and spleen cells with ki67 and DAPI revealed similar G1 fraction sizes in both compartments (Supporting Information S1: Figure 3b); however, we detected increased numbers of S and G2/M cells in the spleen (Figure 3A–C). Of note, the frequency of cells in S/G2/M is higher in both the PB and spleen of *Eµ‐TCL1* than the birthrates described in humans, confirming the aggressive nature of the *Eµ‐TCL1* mouse model. Next, we examined CXCR4 and CD5 expression on leukemic cells in different stages of the cell cycle by overlaying the G0, G1, and S/G2/M fractions, revealing that while G0 and G1 cell fractions displayed similar levels of CXCR4, S/G2/M cells expressed significantly higher levels of both CXCR4 and CD5 (Figure 3D,E) mirroring our findings in human CLL cells.

Leukemic *Eµ‐TCL1* mice present with enlarged spleens.^21^ To investigate if spleen enlargement is associated with the accumulation of cells with distinct phenotypes, we compared CXCR4 and CD5 profiles of spleen and PB CLL cells and observed an expansion of the CXCR4^hi^CD5^hi^ fraction in the spleen, significantly enriched with Ki67+ cells (Supporting Information S1: Figure 3c–e). Altogether, these data suggest that proliferating cells in the *Eµ*‐*TCL1* mouse model have a CXCR4^hi^CD5^hi^ phenotype accumulating predominantly in the spleen.

**Figure 4:**
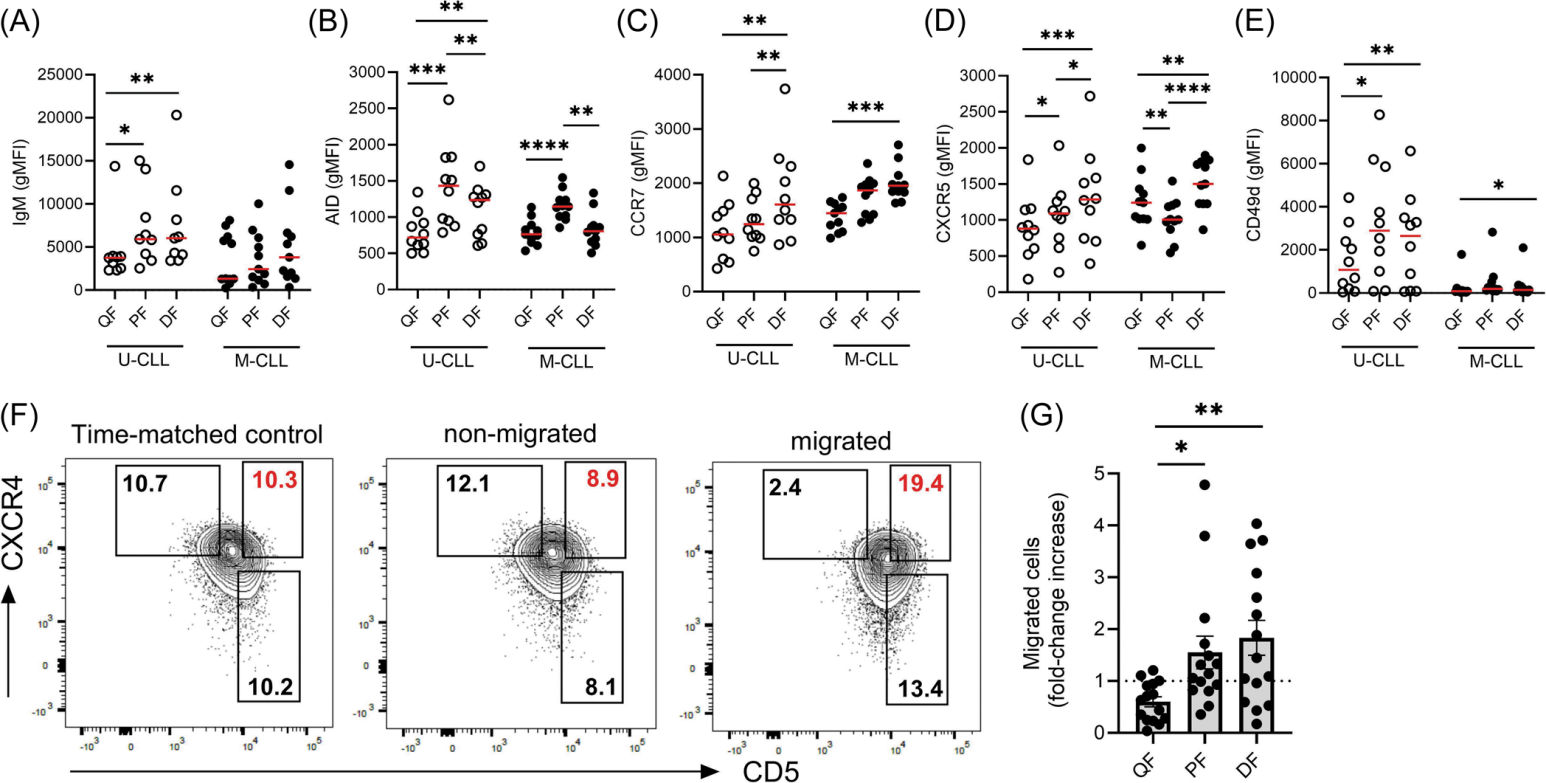
**Proliferating cells have a pro‐migratory phenotype**. Expression levels of **(A)** surface IgM, **(B)** AID, **(C)** CCR7, **(D)** CXCR5, and **(E)** CD49d were quantified on fractions of U‐ and M‐CLL cells. **(F)** PBMCs from U‐CLL patients were placed in transwell migration chambers and incubated in the absence or presence of CCL21. After 2 h cells were harvested from the top chamber (non‐migrated) and bottom chamber (migrated) and cells were stained for CD19, CD5, CXCR4, and CD5. CXCR4 and CD5 gates were drawn on time‐matched controls and extrapolated on migrated and non‐migrated fractions to quantify changing fraction sizes. Representative contour plots are shown from a U‐CLL patient. Fraction percentages are shown within each gate with the changing CXCR4^hi^CD5^hi^ values shown in red. **(G)** Quantification of the fold‐change increase in fraction size in U‐CLL patients of migrated cells in response to CCL21 (1 µg/mL; *n* = 15). Migrated cells, number of migrated cells with chemokine/number of migrated cells in the absence of chemokine. Statistical significance of data was calculated using an RM one‐way ANOVA with Tukey's multiple comparisons *p < 0.05, **p < 0.01, ***p < 0.001, ****p < 0.0001. DF, dividing fraction; PF, proliferated fraction; QF, quiescent fraction.

*Eµ‐TCL1* mice exhibit similar features to aggressive human CLL and the expansion of a Ki67^hi^CXCR4^hi^CD5^hi^ fraction in the spleen may reflect enhanced proliferation rates. Consistent with this idea, examination of human LN cells from a patient with aggressive CLL (de novo TP53‐mutated U‐CLL patient, requiring immediate treatment) revealed a striking expansion of a Ki67^hi^CXCR4^hi^CD5^hi^ fraction in the LN compared to the PB compartment (Supporting Information S1: Figure 4a–c). Conversely, we observed a massively expanded Ki67^hi^PF in the PB while only a negligible PF was detected in the LN, suggesting that the PF in PB may emanate from CXCR4^hi^ populations in tissue. Collectively, these findings support the hypothesis that an expanded CXCR4^hi^CD5^hi^ fraction in tissue may reflect enhanced cell proliferation rates.

### Proliferating CLL cells have a pro‐migratory phenotype

While the PF and QF have been well characterized little is known regarding the phenotype and fate of PB CXCR4^hi^CD5^hi^ cells. In vivo deuterium labeling revealed a correlation between proliferation and high expression of surface IgM,^22^ important for CLL cell re‐stimulation with antigen. We found that IgM was expressed at higher levels on both the DF and PF in U‐CLL patients with the lowest IgM levels on the QF (Figure 4A). In M‐CLL, bulk IgM levels were lower, as expected,^23^ with no significant differences between fractions. Expression of activation‐induced cytidine deaminase (AID) is associated with ki67 expression in tissue CLL cells.^24^ To investigate what fraction(s) express(es) AID we analyzed intracellular AID levels using flow cytometry. AID expression was highest in the PF in both U‐ and M‐CLL patients although in U‐CLL patients the DF expressed higher levels of AID than the QF (Figure 4B).

**Figure 5:**
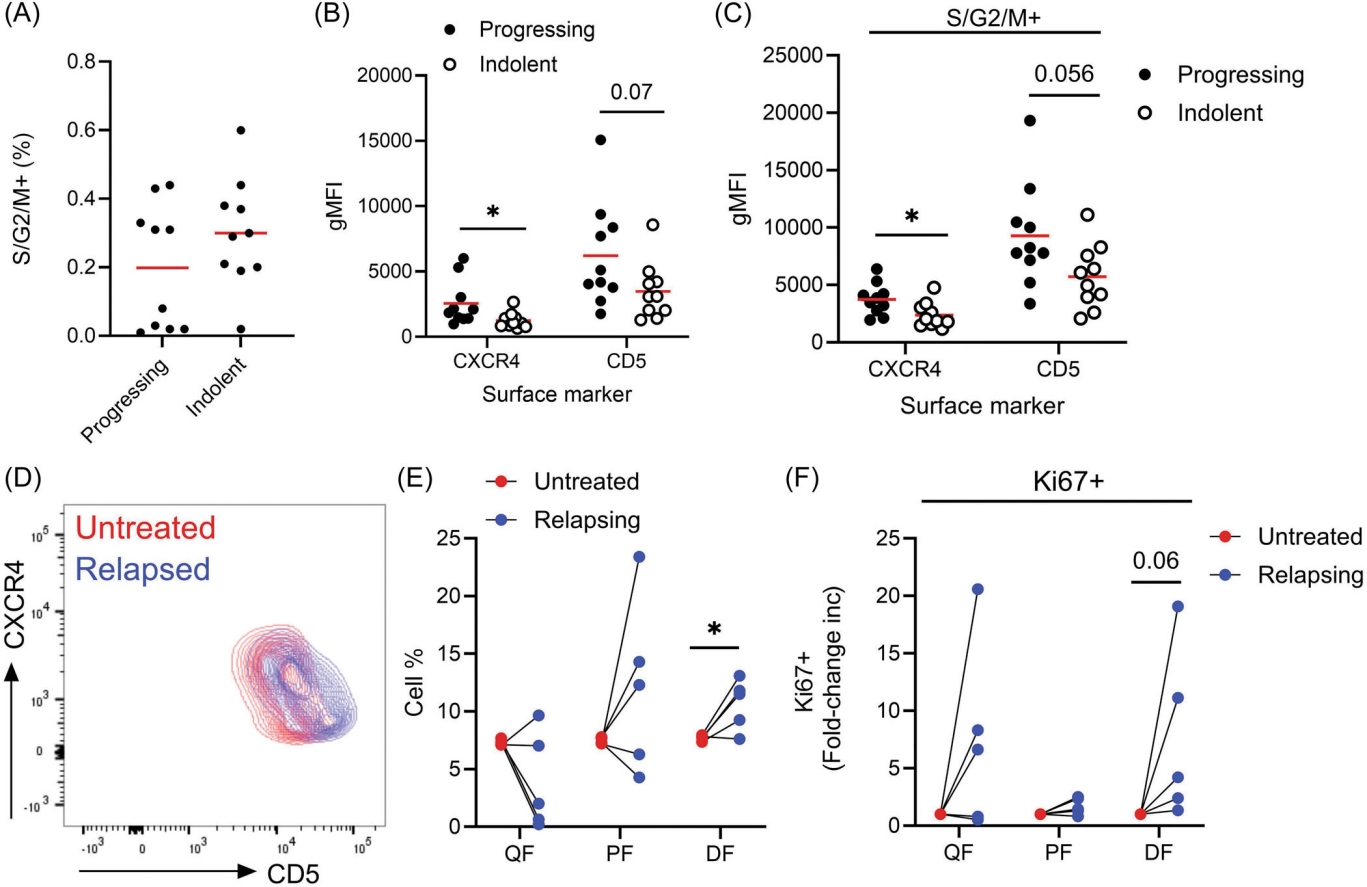
**Disease progression is associated with increased CXCR4 and CD5 levels**. **(A)** PB CLL cells from progressing and indolent untreated cohorts were stained with antibodies against Ki67 and DAPI to quantify S/G2/M fractions. CXCR4 and CD5 levels on PB CLL cells from both progressing and indolent patients were quantified on the **(B)** bulk population and on the **(C)** S/G2/M fraction. Data points represent individual patients. **(D)** CXCR4 and CD5 expression profiles were compared in CLL patients before treatment (red) and at relapse (blue). Representative example plots of a single patient are shown. **(E)** Quantification of cell fraction percentages in five patients prior to treatment (red) and in matched relapsing samples (blue). **(F)** Quantification of the fold‐change increase in the percentage of Ki67+ cells in cell fractions in untreated and matched relapsing samples. Statistical significance of data was calculated using unpaired or paired *t*‐tests where appropriate. *p < 0.05. DF, dividing fraction; PF, proliferated fraction; QF, quiescent fraction.

Previous analysis identified a fraction of CLL cells expressing high levels of IgM and CXCR4,^25^ which may represent a dangerous cell fraction in the PB primed for homing to tissue and receptive to BCR signaling. Thus, we next assessed the expression of receptors important for migration into and positioning within LNs. Strikingly, the DF showed the highest expression of both CCR7 and CXCR5 in both U‐CLL and M‐CLL patients across all fractions assessed (Figure 4C,D) although no difference was observed in bulk levels between U‐CLL and M‐CLL (Supporting Information S1: Figure 5a,b). CD49d was expressed at similar levels on both the PF and DF with the lowest levels observed in the QF (Figure 4E). Thus, CXCR4^hi^CD5^hi^ cells express increased levels of both chemokine and adhesion receptors.

CCR7 has been strongly implicated in LN homing^26^ and we next assessed whether higher levels of CCR7 on CXCR4^hi^CD5^hi^ cells confer a greater propensity to migrate toward CCL21. When examining the bulk population, U‐CLL cells migrated more efficiently in transwell assays than M‐CLL cells at higher concentrations of CCL21, while no differences were detected at lower CCL21 concentrations (Supporting Information S1: Figure 5c). To assess which cell fraction migrated most efficiently toward CCL21, we initially attempted to sort QF, PF, and DFs; however, bulk CXCR4 and CD5 levels fluctuated, indicating that the sorting process induced phenotypic changes. Subsequently, we used time‐matched controls, incubated in the presence of CCL21, to draw gates capturing 10% of the bulk population for the QF, PF, and DF to account for fluctuations in CXCR4 and CD5 induced by in vitro incubation (Figure 4F, left panel). Importantly, both CXCR4 and CD5 expression levels did not fluctuate in response to cells binding to either CCL21 or ICAM‐1 (Supporting Information S1: Figure 5d–g). Migrated cells were significantly enriched with cells from the DF and PF compared to QFs in U‐CLL samples (1.8 ± 1.3, 1.5 ± 1.2, and 0.6 ± 0.3 fold‐change fraction increases, respectively; Figure 4F,G). We observed significant enrichment of both the PF and DF in migrated cells in response to CCL21 while conversely, cells with a QF phenotype significantly declined with increasing concentrations of CCL21 (Supporting Information S1: Figure 5h). Contrastingly, in M‐CLL samples, the PF showed the largest expansion, while a simultaneous increase in cells from the DF failed to reach statistical significance (*p* = 0.08; Supporting Information S1: Figure 5i). Altogether these data highlight the DF in PB as a cell fraction with heightened migratory potential toward CCL21, expressing increased levels of IgM, CD49d, CCR7, CXCR5, and CXCR4, which may facilitate LN homing and re‐stimulation.

### Disease progression is associated with increased CXCR4 and CD5 levels

To investigate whether the presence of proliferating cells in the periphery is indicative of proliferation rates in tissue, we next assessed whether patients with progressing disease show larger S/G2/M cell fractions in the PB compared to more indolent patients. Surprisingly, no differences were detected in the size of the S/G2/M between an indolent and a progressing cohort (*p* = 0.361), suggesting that irrespective of disease state the fraction of dividing cells in the blood remains constant (Figure 5A). Consistent with CXCR4 being a negative prognostic marker in CLL,^27^ we observed higher expression levels of surface CXCR4 on the bulk population of progressing patients and a trend for higher levels of CD5 (*p* = 0.07; Figure 5B). S/G2/M cells in progressing patients expressed higher levels of CXCR4 compared to S/G2/M cells in indolent patients with a strong trend for increased expression of CD5 (*p* = 0.056; Figure 5C). We next assessed whether changes in the DF occur within patients progressing over time rather than comparing between patients. Setting gates to capture the QF, PF, and DF, we compared fraction sizes in PB CLL samples from patients relapsing on treatment to samples taken prior to treatment. We observed expansion of the DF in all five patients when relapsing (Figure 5D,E), with a trend for increased Ki67 expression in the DF at relapse (*p* = 0.06) (Figure 5F). Altogether, we conclude that an expansion of the CXCR4^hi^CD5^hi^ fraction reflects higher net cell birth rates and disease progression.

## DISCUSSION

Deuterium incorporation studies highlighted both quiescent and newly born recent LN emigrants in the PB; however, identifying actively dividing cells has proven challenging. Here, we combined DNA labeling with Ki67 staining to distinguish cells in G0, G1, and S/G2/M phases and found a small fraction of CLL cells in S/G2/M phase readily detectable in the PB of all patients, regardless of *IGHV* mutation status or disease course. On average, 0.24% ± 0.17% of CLL cells were in the S/G2/M phase, a value surprisingly consistent with previous estimations for daily tumor proliferation rates of 0.1%–1%^1^ and 0.2%^22^ and not much lower than recently reported for LN CLL cells (0.4%–1%).^10^ As CLL patients have ≥5 × 10^9^ CLL cells/L in the periphery, the proliferation of 0.2% PB CLL cells may represent a substantial part of cell turnover.

Recent single‐cell transcriptomic analysis of both LN and PB CLL cells distinguished three major cell states, quiescent, activated, and proliferating,^10^ while the CXCR4/CD5 model describes recently divided and quiescent cell fractions.^6^ Adding to these studies, our data demonstrate that actively dividing CLL cells uniformly express high levels of both CXCR4 and CD5, indicating that the CXCR4^hi^CD5^hi^ fraction contains actively proliferating cells, while the PF contains few cells in S/G2/M‐phase. Likely, the higher enrichment of ^2^H‐labeled DNA in the PF reflects proliferation histories rather than active proliferation. Of note, the CXCR4^hi^CD5^hi^ fraction contained ^2^H‐labeled cells in vivo^28^ in agreement with our observation of mitotic cells in this fraction. Separately, in vivo pulse‐chase labeling with deuterated glucose revealed a correlation between proliferation and high IgM levels,^22^ consistent with our finding of high IgM levels in the DF. Previous studies labeled the PF as a proliferating fraction based on high Ki67 expression.^5^, ^6^ However, the ability of Ki67 to discriminate actively dividing cells is debated.^29^, ^30^ While absent in G0 cells,^31^ Ki67 messenger RNA and protein levels increase in G1 cells that have not (yet) committed to the cell cycle.^32^, ^33^ Based on our data we conclude that the PF is enriched in G1 cells. Indeed, proteins regulating the G1‐S transition, such as *Cyclin E1, Cyclin D2*, and MCM6^34^, ^35^, ^36^ are upregulated in the PF.^6^

Among PB CXCR4^lo^CD5^hi^ CLL cells, only a minor subfraction of activated cells in G1 may progress to the proliferating state where the life cycle of a single CLL cell starts over, while the majority of the CXCR4^lo^CD5^hi^ cell fraction may exit to G0. This is in line with recent RNA velocity analysis of single‐cell transcriptomics data, indicating that LN CLL cells transverse unidirectionally from proliferating to an activated state before entering a final quiescent state.^10^ To examine fates after cell division, we tracked proliferation histories in vitro, revealing that by Day 9 the divided fraction contained cells with both quiescent and proliferating phenotypes, indicating that cell division may give rise to daughter cells with distinct fates. Based on our observation that the PF can be absent in the LN of a CLL patient with aggressive disease and expanded in the PB, we propose that stimulation of CXCR4^hi^ cells in tissue leads to downregulation of CXCR4 and LN egress, giving rise to the CXCR4^lo^ PF phenotype in the PB. Subsequent transition of cells from the PF to the DF is likely dependent on the strength and nature of the initial stimulation with only highly stimulated cells progressing to S/G2/M phases and the remaining cells transitioning to more quiescent phenotypes. Reconciling these studies and our data, we postulate that CXCR4^lo^CD5^hi^ cells are enriched for recently divided (re‐)activated recent LN emigrant cells that are in G1 at the time of sampling, while CXCR4^hi^CD5^hi^ cells are enriched in actively dividing cells in S/G2/M phase and CXCR4^hi^CD5^lo^ cells are predominantly in a quiescent state.

Additionally, we detected an expanded Ki67^hi^ DF in the LN of a patient with aggressive disease and observed a similar phenomenon in the spleens of *Eµ‐TCL1* mice that develop an aggressive CLL‐like disease, suggesting that an expanded DF in tissue is indicative of increased cell turnover. Interestingly, the PF phenotype could not be detected in the PB or SP of the *Eµ‐TCL1* mouse model, suggesting that the CXCR4/CD5 kinetics model described for human CLL may not be recapitulated fully in this mouse model. Conversely, we observed a decreased frequency of Ki67+ cells in the BM of *Eµ‐TCL1* mice, consistent with human data where the BM compartment shows reduced proliferative capacity.^5^, ^37^

While high CD5 expression may reflect recent activation, our observation that CXCR4 levels rise gradually from G1 to G2/M establishes a strong link between CXCR4 expression and cell cycle progression in CLL cells. Cell cycle entry may influence CXCR4 dynamics as CXCR4 internalization is detected 2 h post BCR stimulation in CLL cells,^38^ consistent with lymphocytes committing to the G1 stage 3 h post a stimulatory event.^39^ Subsequent CXCR4 upregulation on dividing cells likely requires the synthesis of new CXCR4 transcripts as the majority of intracellular CXCR4 levels are degraded ~6 h poststimulation,^38^ in contrast to rapid recovery of CXCR4 expression in the absence of stimulation due to recycling of the receptor.^25^, ^40^ Fluctuations in CXCR4 expression on proliferating CLL cells may mirror the changes in CXCR4 levels on GC B cells where CXCR4 expression discriminates between proliferating and nonproliferating subsets in the dark (DZ) and light zones (LZ), respectively.^41^, ^42^ In support, and consistent with previous analysis,^24^ we observe the highest levels of AID in the PF in CLL, enriched in G1 cells, analogous to restricted AID activity in early G1‐phase B cells in the DZ.^43^ Finally, GC B cells are most sensitive to BCR stimulation in the G2/M phase,^44^ while we find that the DF expresses high levels of IgM, associated with more efficient BCR signaling.^25^ Direct regulation of the cell cycle by CXCR4 has yet to be established in CLL although previous studies have implicated CXCR4 signaling in cell cycle regulation in solid tumors. CXCR4 silencing inhibits cell growth in human carcinoma cell lines^45^ and increased expression of CXCR4 in G2/M phases has been reported in breast cancer cell lines where proteomic analysis revealed crosstalk between CXCR4 and G2‐M transition proteins, such as PLK1 and Cyclin B1.^46^ This suggests that rather than a surrogate marker for cell division, active CXCR4 signaling in CLL cells may be an important regulator of the mitotic process.

We detected no significant differences in the frequency of S/G2/M cells between progressing and indolent cases suggesting enhanced PB‐LN recirculation dynamics in progressing patients. Furthermore, the CXCR4^hi^CD5^hi^ phenotype of mitotic cells may be highly transient with cells either rapidly returning to tissue for restimulation or downregulating CD5 to transition to the QF where they accumulate and die. Supportive of the first scenario, we detected efficient migration toward CCL21, required for LN homing, on both the DF and PF although in our experiments we cannot exclude that the migration process itself does not impact CXCR4 and CD5 expression levels. Alternatively, the accumulation of quiescent CXCR4^hi^CD5^hi^ cells over time may obscure the DF, limiting its use as a prognostic indicator. Expansion of the PF has been recently reported to be associated with clinical relapse.^47^ Accordingly, we detected expanded PFs in 3/5 patients. Importantly, we consistently detected increased sizes of the DF in all patients at relapse compared to matched untreated samples, indicating that tracking the outgrowth of the DF may serve as an early biomarker of clinical progression. Moreover, as genetic mutations likely arise during the S phase, sequencing of cells in the G2/M phase may reveal the acquisition of new genomic alterations, as well as the genetics of the most proliferative clones.

Thus, recent activating events that give rise to activated/proliferating cell fractions in the PF/DF may underlie the migratory differences we observed between the fractions. Previous analysis alluded to a minor subfraction of PB CLL cells expressing high levels of CXCR4 and IgM, open to both migration to tissue and BCR signals.^25^ Cells in the DF are strong candidates for this subpopulation where divided cells are uniquely poised to home to tissue for restimulation. Importantly, their expression of CXCR4, CCR7, CXCR5, CD49d, and IgM suggest these cells can respond to a range of tissue chemokine gradients maximizing cell‐cell interactions.^48^, ^49^ Our findings may facilitate therapeutic strategies targeting the most dangerous CLL cell fractions and provide a strong rationale for the use of monoclonal antibodies for CCR7 and CXCR4 that are currently being trialed.^50^, ^51^

## AUTHOR CONTRIBUTIONS

Daniel Friedman conceptualized and designed the study, performed experiments, analyzed data, and wrote the manuscript. Drshika P. Mehtani performed experiments and analyzed data. Jennifer B. Vidler performed experiments. Piers E. M. Patten designed the study and wrote the manuscript. Robbert Hoogeboom conceptualized and designed the study, supervised the project, and wrote the manuscript.

## CONFLICT OF INTEREST STATEMENT

The authors declare no conflict of interest.

## ETHICS STATEMENT

This research was supported by material from the King's College Denmark Hill Haematology Biobank, ethics approval 18/NE/0141. Lymph node fine needle aspirates and cryopreserved peripheral blood samples were obtained from CLL patients with written informed consent in accordance with the Declaration of Helsinki.

## FUNDING

Piers E. M. Patten received research funding from Roche and Gilead and honoraria from Abbvie, Astra Zeneca, Beigene, Gilead, and Janssen. This work was supported by grants from Leukaemia UK (2019/JGF/002), Blood Cancer UK (22006) (RH), and the Lymphoma Research Trust (LRT6147) (DF).

## Supporting information

Supporting information.

## Data Availability

The data that support the findings of this study are available from the corresponding author upon reasonable request.
