# Supplementary material for: Proliferating CLL cells express high levels of CXCR4 and CD5
Source: Hemasphere. 2024 Dec 17;8(12):e70064. doi: 10.1002/hem3.70064 (PMC11651208; doi:10.1002/hem3.70064)
Supplement: Supplementary file 1 — Supporting information. [file HEM3-8-e70064-s001.docx]

**Supplementary table 1. Demographic and clinical characteristics of patients**. IGHV, immunoglobulin heavy-chain gene; M, mutated; U, unmutated; PT, pre-treatment; R, relapsing; NK, not known.

| Patient ID | Age | Gender | IGHV | Cytogenetic abnormalities | Figures |
| --- | --- | --- | --- | --- | --- |
| 0201 | 66 | M | M | None detected | 1,5 |
| 0212 | 62 | F | M | None detected | 1,5 |
| 0140 | 84 | F | U | del(11q) | 1,2,4,5 |
| 0607 | 65 | M | U | del(17p) | 1,4,5 |
| 0614 | 49 | M | U | del(11q) | 1,5 |
| 0785 | 59 | M | M | del(13q) | 1,5 |
| 0007 | 55 | M | M | del(13q) | 1,5 |
| 0299 | 77 | M | U | del(17p) | 1,2,4,5 |
| 1966 | 74 | M | M | del(13q) | 1,5 |
| 2537 | 67 | M | M | del(13q) | 1,5 |
| 0018 | 59 | M | U | del(13q) | 1,2,4,5 |
| 2563 | 51 | M | U | None detected | 1,5 |
| 0105 | 76 | M | U | None detected | 1,5 |
| 0079 | 27 | M | U | del(11q) | 1,5 |
| 0244 | 61 | M | U | Trisomy 12 | 1,5 |
| 0259 | 69 | F | M | None detected | 1,5 |
| 0287 | 57 | F | M | del(13q) | 1,5 |
| 0159 | 69 | M | M | None detected | 1,5 |
| 0050 | 58 | M | M | del(11q), trisomy 12 | 1,5 |
| 0273 | 72 | F | U | del(13q) | 1,2,4,5 |
| 0363 | 65 | M | U | del(17p) | 2 |
| 0342 | 70 | F | U | None detected | 2,4 |
| 0365 | 62 | M | U | del(11q) | 2,4 |
| 0030 | 39 | M | U | del(17p), complex karyotype | 2 |
| 0161 | 63 | M | U | del(13q) | 2,4 |
| 0025 | 44 | M | U | None detected | 2,4 |
| 0230 | 62 | M | U | del(17p) | 2 |
| 0008 | 68 | M | U | del(17p) | 2,4 |
| 0017 | 63 | M | U | del(13q) | 4 |
| 0040 | 67 | M | U | None detected | 4 |
| 0081 | 46 | M | U | del(11q) | 4 |
| 0168 | 72 | M | U | None detected | 4 |
| 0190 | 80 | M | U | del(11q) | 4 |
| 0776 | 53 | M | U | del(11q) | 4 |
| 0125 | 81 | F | U | del(17p) | 4 |
| 0257 | 77 | M | U | None detected | 4 |
| 0016 | 83 | M | U | del(17p) | 4 |
| 0356 | 62 | M | U | del(11q) | 4 |
| 0151 | 58 | M | M | del(13q) | 4 |
| 0152 | 60 | M | M | Trisomy 12 | 4 |
| 0160 | 63 | F | M | del(13q) | 4 |
| 0016 | 67 | M | M | None detected | 4 |
| 0121 | 62 | F | M | None detected | 4 |
| 0267 | 65 | M | M | del(13q) | 4 |
| 0353 | 46 | F | M | del(13q) | 4 |
| 0003 | 77 | F | M | None detected | 4 |
| 0102 | 73 | F | M | del(13q) | 4 |
| 0298 | 46 | M | M | None detected | 4 |
| 0742-PT | 58 | M | M | ATM del (11q/13q) | 5 |
| 0742-R | 61 | M | M | ATM del (11q/13q), BTK Cys481Ser | 5 |
| 0173-PT | 32 | F | U | del(11q) | 5 |
| 0173-R | 40 | F | U | del(17p), BTK Cys481Arg | 5 |
| 0244-PT | 61 | M | U | del(13q) | 5 |
| 0244-R | 64 | M | U | del(13q) | 5 |
| 0046-PT | 49 | M | NK | None detected | 5 |
| 0046-R | 50 | M | NK | del(13q) | 5 |
| 0259-PT | 61 | M | U | del(17p/11q) | 5 |
| 0259-R | 63 | M | U | del(17p/11q) | 5 |

**Supplementary Figure 1.**


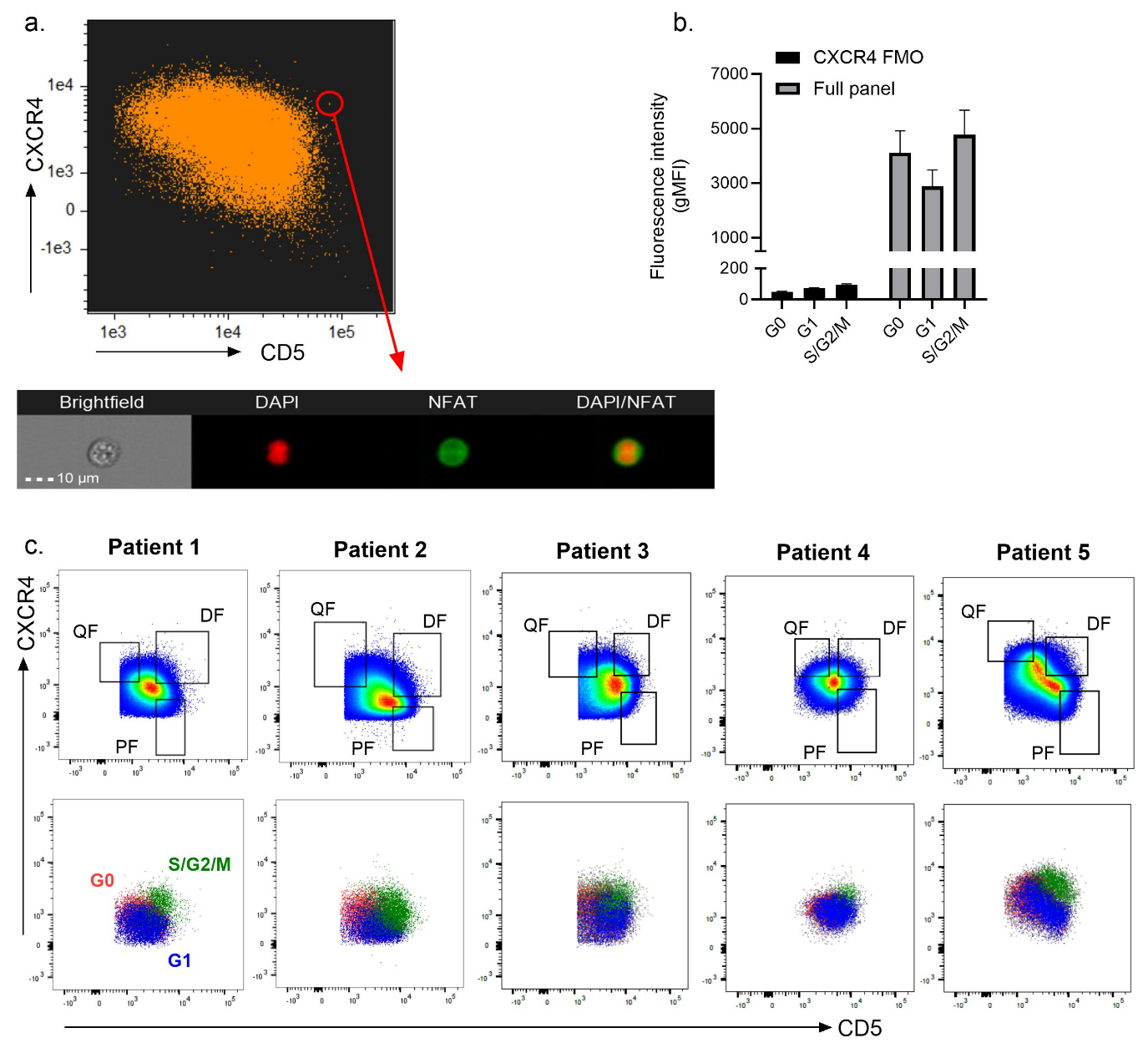


**Supplementary figure 1. Proliferating CLL cells can be observed in the CXCR4^hi^CD5^hi^ fraction.** (a) Image of a dividing CXCR4^hi^CD5^hi^ PB CLL cell in G2/M phase acquired using imaging flow microscopy. Red: DAPI (nucleus); Green: NFAT2 (cytoplasm). PB, peripheral blood. Images shown are representative of four independent patients. (b) Flow cytometry was used to quantify the fluorescence intensities of G0/G1 and S/G2/M cell fractions in the absence (fluorescence minus one) or presence of a CXCR4 fluorescent antibody. n=4. Data shows mean±SD (c) Representative CXCR4/CD5 contour plots obtained using flow cytometry from 5 CLL patients demonstrating the gating strategy for the quiescent fraction (QF), proliferated fraction (PF) and the dividing fraction (DF); (upper panels) with matching CXCR4/CD5 plots with G0 (red), G1 (blue) and S/G2/M (green) populations overlaid (lower panels). Gates were set to capture 5% of the bulk population in each fraction.

**Supplementary Figure 2.**


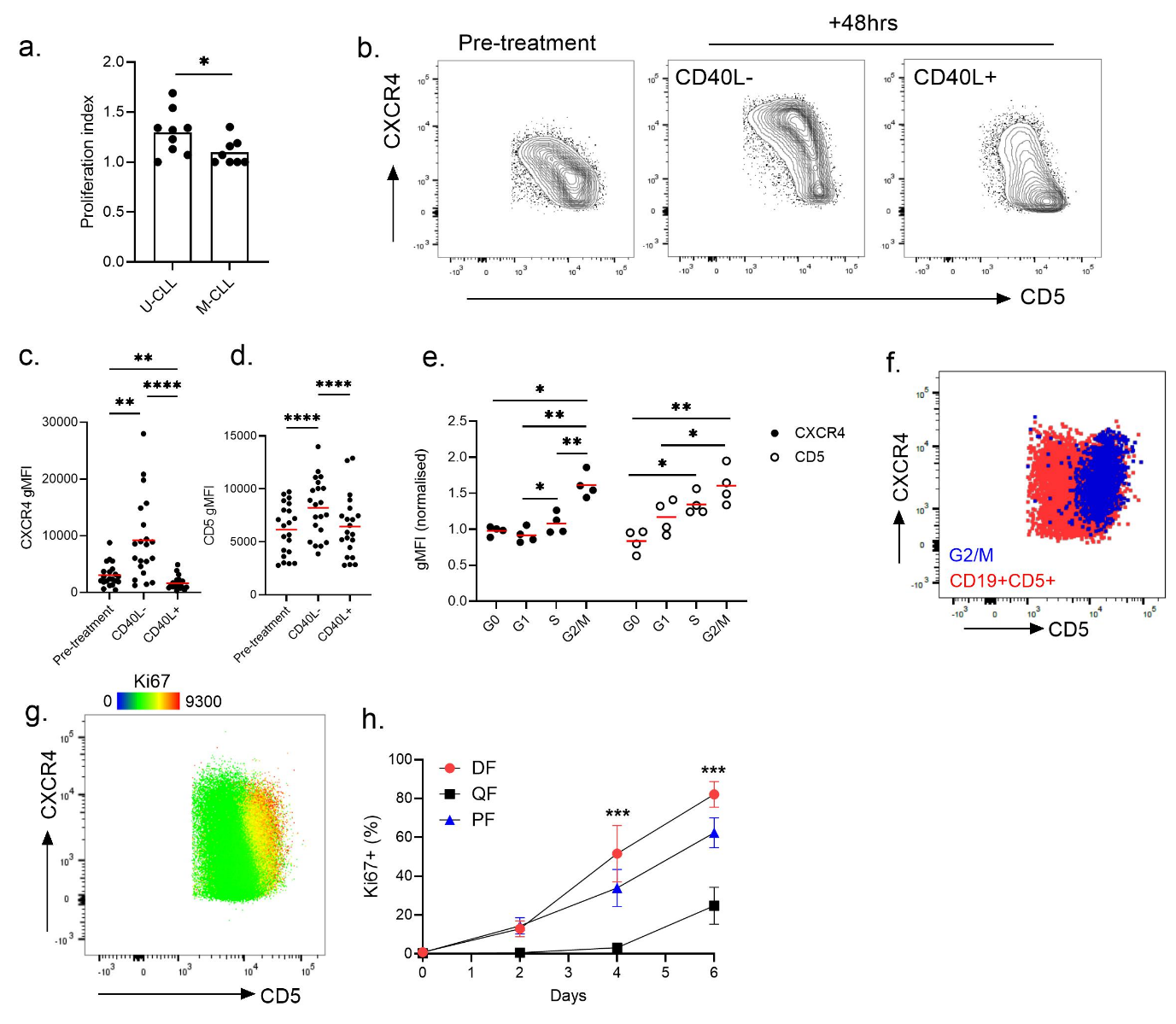


**Supplementary figure 2. CXCR4 expression is downregulated in response to activating signals.** (a) Proliferation indices of U-CLL and M-CLL cells stimulated on CD40L+ fibroblasts for 9 days (U-CLL n=10, M-CLL n=9). (b) Human U-CLL PBMCs were seeded on CD40L-, CD40L+ fibroblasts for 48 hrs and stained for CD19, CD5, and CXCR4. Representative contour CXCR4/CD5 plots from cells prior to treatment and after 48hrs in different conditions are shown. (c) Bulk CXCR4 and (d) CD5 levels were quantified on CLL cells both prior to treatment and after 48hrs stimulation Data points represent individual patients. (n=21). (e) Quantification of CXCR4 and CD5 expression levels of U-CLL cells in G0, G1, S, and G2/M phases when stimulated on CD40L+ fibroblasts plus Interleukin (IL) 4 and IL-21 after 6 days. Data were normalised to the bulk CXCR4/CD5 gMFI levels. (n=4). (f) Representative CXCR4/CD5 scatter plot overlaid with the G2/M fraction from U-CLL cells stimulated on CD40L+ fibroblasts and IL-4 and IL-21 for 6 days. (g) Human U-CLL cells were seeded on CD40L+ fibroblasts plus IL-4 and IL-21 for 6 days and samples assessed for Ki67 expression on days 0, 2, 4 and 6. Representative scatter plots from a U-CLL patient of CXCR4 and CD5 profiles from day 4 are shown with Ki67 gMFIs overlaid as a heatmap statistic. (h) Frequency of Ki67+ cells in different cell fractions of U-CLL cultured over 6 days (n=8). gMFI, geometric mean fluorescence intensities. Statistical significance of data were calculated using a RM one way ANOVA with Tukey’s multiple comparisons or unpaired t-tests. *p <0.05 **p <0.01, ***p <0.001 ****p <0.0001.

**Supplementary Figure 3.**

**
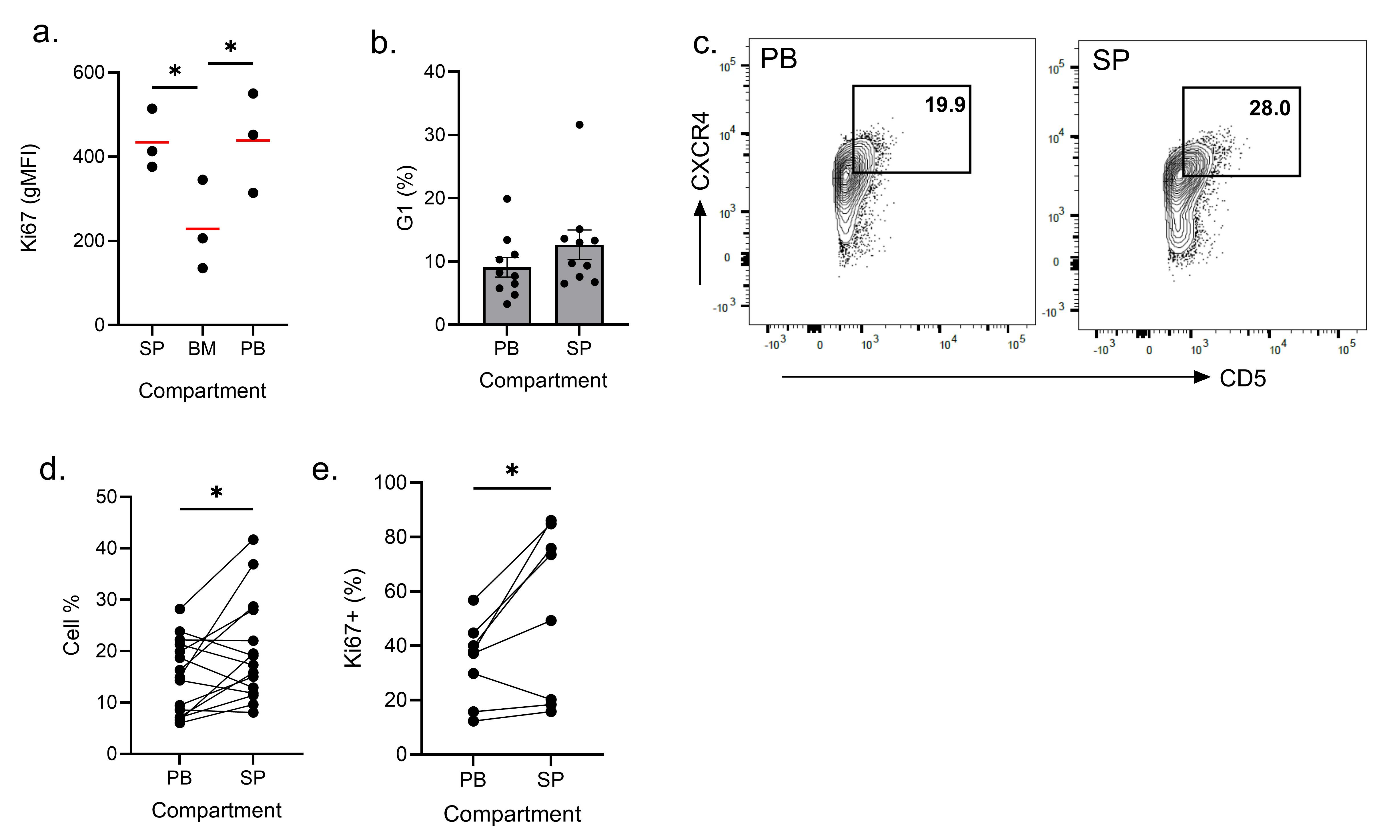
**

**Supplementary figure 3. An expanded Ki67^hi^CXCR4^hi^CD5^hi^ fraction is detected in murine tissue.** (a) Quantification of Ki67 levels in CD19+CD5+ TCL1 cells from the spleen (SP), bone marrow (BM) and peripheral blood (PB). Each data point represents a single mouse (n=3). (b) Quantification of the percentage of cells in G1 between PB and SP compartments. (n=9) (c) Representative contour plots comparing CXCR4/CD5 profiles in cells from the PB and spleen. Gates were drawn on the PB and extrapolated onto spleen cell plots. Quantification of (d) CXCR4^hi^CD5^hi^ cell percentages (n=15) and (e) Ki67+ cells (n=8) in the CXCR4^hi^CD5^hi^ fraction in both PB and SP. Statistical significance of data were calculated using paired t-tests. *p <0.05.

**Supplementary Figure 4.**

**Supplementary figure 4. An expanded CXCR4^hi^CD5^hi^ fraction is observed in the lymph nodes of a CLL patient with aggressive disease.** (a) CXCR4 and CD5 contour plots of lymph node (LN) and matched peripheral blood (PB) CLL cells from a de novo U-CLL patient with mutated TP53 and rapidly progressing disease. (b) CXCR4 and CD5 contour plots with Ki67 gMFIs overlaid as a heatmap statistic. (c) Quantification of intracellular Ki67 with colours reflecting the different cell fractions gated in (a). gMFI, geometric mean fluorescence intensities. QF: Quiescent Fraction, PF: Proliferated Fraction, DF: Dividing Fraction.


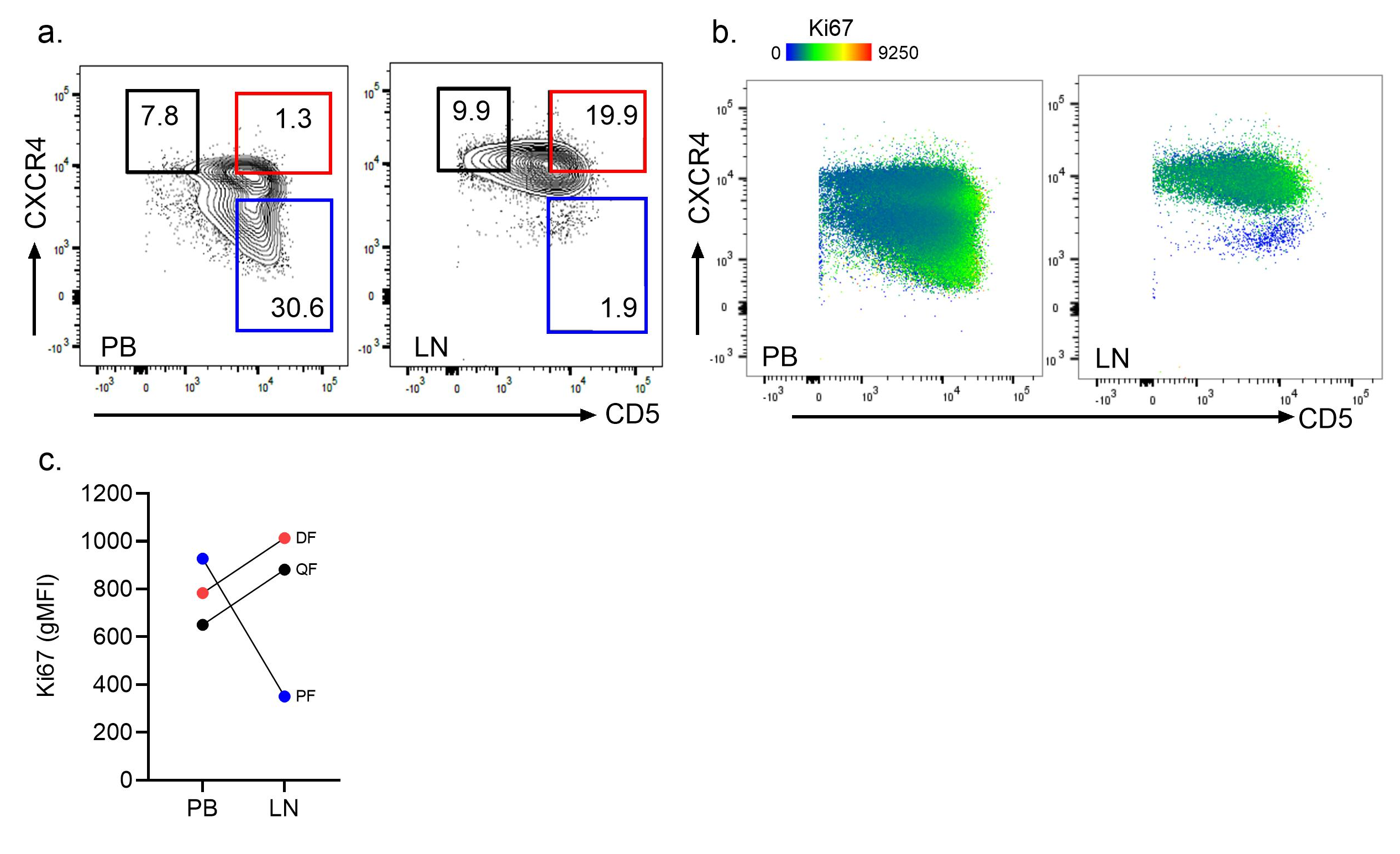


**Supplementary Figure 5.**


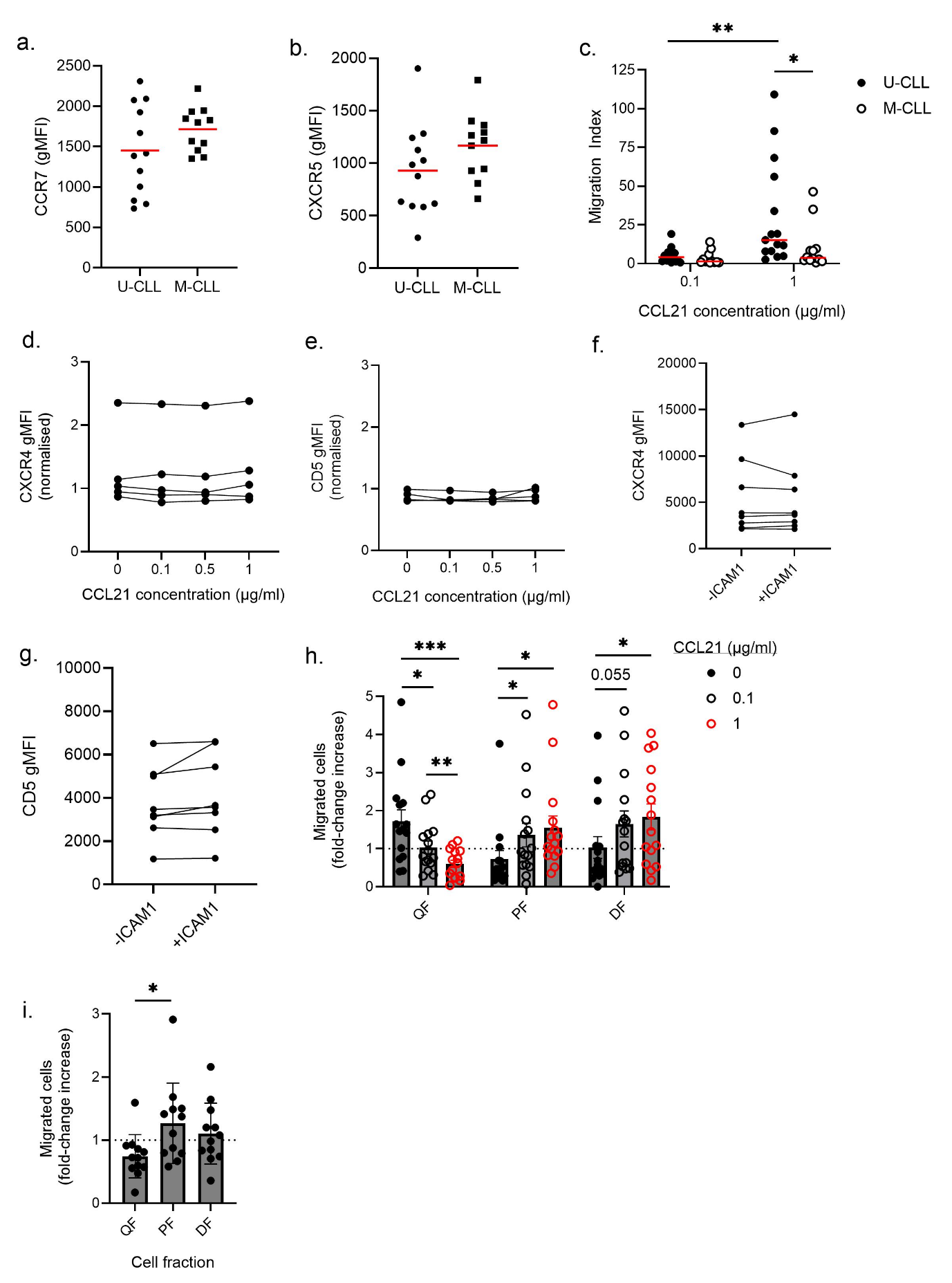


**Supplementary figure 5. CXCR4 and CD5 levels do not fluctuate in response to CCR7 signalling.** Bulk expression levels of (a) CCR7 and (b) CXCR5 were quantified on U- and M-CLL cells. (c) PBMCs from both U- and M-CLL patients were placed in transwell migration chambers and incubated in the absence or presence of increasing concentrations of CCL21. After 2hrs, migrated cells were stained for CD19 and CD5 and numbers quantified by flow cytometry. Migration index = number of migrated cells with chemokine/number of migrated cells in the absence of chemokine. (d) PBMCs were incubated with increasing concentrations of CCL21 for 2hrs at 37°C and CXCR4 and (e) CD5 levels quantified by flow cytometry (n=5). (f) PBMCs were seeded on ICAM-1 coated well plates for 2hrs at 37°C and CXCR4 and (g) CD5 levels quantified by flow cytometry (n=8). (h) PBMCs from U-CLL patients were placed in transwell migration chambers and incubated for 2hrs at 37°C in the absence or presence of increasing concentrations of CCL21. After 2hrs migrated cells were harvested from the bottom chamber and stained with antibodies against CD19, CD5, CXCR4 and CD5. CXCR4 and CD5 gates were drawn on time matched controls and extrapolated on migrated fractions to quantify changing fraction sizes (n=15). Data points represent individual patients. (i) Quantification of the fold-change increase in fraction size in M-CLL patients of migrated cells in response to CCL21 (1µg/ml) n=12. Migrated cells = number of migrated cells with chemokine/number of migrated cells in the absence of chemokine. QF: Quiescent Fraction, PF: Proliferated Fraction, DF: Dividing Fraction. Statistical significance of data were calculated using a RM one way ANOVA with Tukey’s multiple comparisons or an unpaired parametric t-test. *p <0.05, **p <0.01, ***p<0.001.
